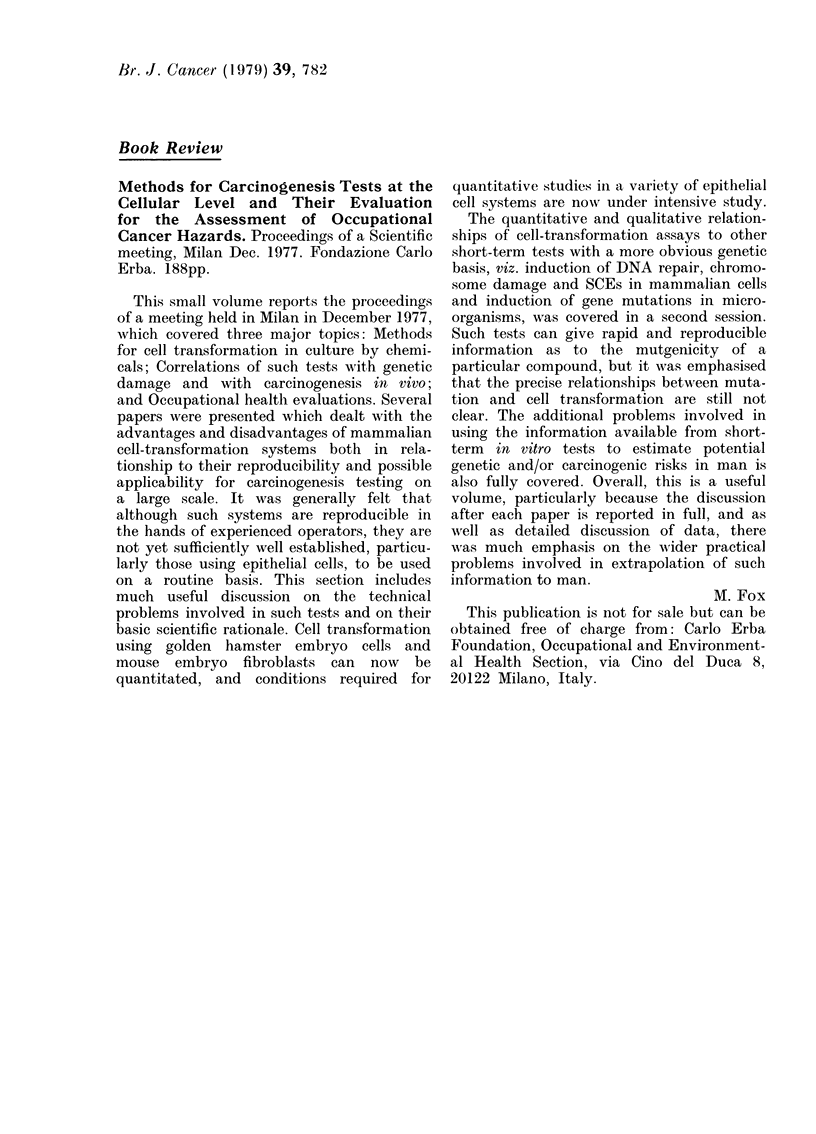# Methods for Carcinogensis Tests at the Cellular Level and Their Evaluation for the Assessment of Occupational Cancer Hazards

**Published:** 1979-06

**Authors:** M. Fox


*Br. J. Cancer* (1979) **39**, 782

### *Book Review*

**Methods for Carcinogenesis Tests at the Cellular Level and Their Evaluation for the Assessment of Occupational Cancer Hazards.** Proceedings of a Scientific meeting, Milan Dec. 1977. Fondazione Carlo Erba. 188pp.

This small volume reports the proceedings of a meeting held in Milan in December 1977, which covered three major topics: Methods for cell transformation in culture by chemicals; Correlations of such tests with genetic damage and with carcinogenesis *in vivo*; and Occupational health evaluations. Several papers were presented which dealt with the advantages and disadvantages of mammalian cell-transformation systems both in relationship to their reproducibility and possible applicability for carcinogenesis testing on a large scale. It was generally felt that although such systems are reproducible in the hands of experienced operators, they are not yet sufficiently well established, particularly those using epithelial cells, to be used on a routine basis. This section includes much useful discussion on the technical problems involved in such tests and on their basic scientific rationale. Cell transformation using golden hamster embryo cells and mouse embryo fibroblasts can now be quantitated, and conditions required for quantitative studies in a variety of epithelial cell systems are now under intensive study.

The quantitative and qualitative relationships of cell-transformation assays to other short-term tests with a more obvious genetic basis, *viz.* induction of DNA repair, chromosome damage and SCEs in mammalian cells and induction of gene mutations in microorganisms, was covered in a second session. Such tests can give rapid and reproducible information as to the mutgenicity of a particular compound, but it was emphasised that the precise relationships between mutation and cell transformation are still not clear. The additional problems involved in using the information available from short-term *in vitro* tests to estimate potential genetic and/or carcinogenic risks in man is also fully covered. Overall, this is a useful volume, particularly because the discussion after each paper is reported in full, and as well as detailed discussion of data, there was much emphasis on the wider practical problems involved in extrapolation of such information to man.

M. Fox

This publication is not for sale but can be obtained free of charge from: Carlo Erba Foundation, Occupational and Environmental Health Section, via Cino del Duca 8, 20122 Milano, Italy.